# Myofascial pain in older adults: a geroscience-informed framework integrating precision geriatrics and digital therapeutics

**DOI:** 10.3389/fnagi.2026.1806386

**Published:** 2026-04-16

**Authors:** Chang Zhu, Jie Xu, Yating Gong, Chaoyu Wang, Wenting Xu, Lijun Chen

**Affiliations:** 1 Department of Rehabilitation, Zhangjiagang TCM Hospital Affiliated to Nanjing University of Chinese Medicine, Zhangjiagang, Jiangsu, China; 2 Department of General Medicine, Zhangjiagang TCM Hospital Affiliated to Nanjing University of Chinese Medicine, Zhangjiagang, Jiangsu, China; 3 Department of Reproduction, Zhangjiagang TCM Hospital Affiliated to Nanjing University of Chinese Medicine, Zhangjiagang, Jiangsu, China

**Keywords:** biopsychosocial model, central sensitization, digital therapeutics, elderly, geroscience, myofascial pain syndrome

## Abstract

Myofascial pain syndrome (MPS) in the elderly is a significant contributor to chronic pain and functional decline, driven by a unique set of age-related pathophysiological challenges. Core geroscience principles—including sarcopenia, inflammaging, and cellular senescence—converge to create a state of heightened central sensitization that amplifies pain. Traditional pharmacotherapy is fraught with risks in this population due to polypharmacy and vulnerability to adverse effects, with many common analgesics discouraged by geriatric guidelines. This review proposes an innovative biopsychosocial framework integrating precision geriatrics and digital therapeutics to address these challenges. Precision geriatrics employs multidimensional assessment tools (e.g., ultrasonography, frailty indices) to phenotype patients and tailor interventions, while digital therapeutics deliver evidence-based non-pharmacological interventions like cognitive behavioral therapy and therapeutic exercise, overcoming access barriers. The proposed framework prioritizes non-pharmacological strategies, advocates for structured deprescribing, and emphasizes geriatrically-adapted interventions delivered through multidisciplinary teams. By synthesizing recent evidence, this review provides a comprehensive roadmap for clinicians to move beyond a one-size-fits-all approach, offering safer, more effective care that improves function, independence, and quality of life for older adults with MPS.

## 1 Introduction: the “hidden epidemic” of myofascial pain in the elderly

Chronic pain is a pervasive public health issue, disproportionately affecting older adults. It is estimated that over 50% of community-dwelling older adults and up to 80% of those in long-term care facilities suffer from chronic pain (Ge et al., 2025; Pasiri et al., 2025), which profoundly impairs physical function, diminishes quality of life, and exacerbates comorbidities such as depression and cognitive decline (Tinnirello et al., 2021; Honghong et al., 2025). Recent epidemiological data underscore the magnitude of this challenge. According to the 2023 National Health Interview Survey, 24.3% of U. S. adults experience chronic pain, with prevalence increasing significantly with age (Lucas and Sohi, 2024). Myofascial pain syndrome represents a substantial proportion of this burden. A 2024 comprehensive review identified MPS as a major player in musculoskeletal pain, affecting up to 85% of individuals with chronic pain at some point in their lives (Lam et al., 2024). In geriatric populations, the prevalence may be even higher due to age-related changes in muscle tissue, fascia, and pain processing mechanisms (Steen et al., 2025). Among the myriad causes of chronic pain in this population, Myofascial Pain Syndrome (MPS) represents a significant and frequently under-recognized contributor. MPS is a non-articular musculoskeletal condition characterized by the presence of myofascial trigger points (MTrPs)—hyperirritable nodules located within a taut band of skeletal muscle that are painful upon compression and can give rise to characteristic referred pain, motor dysfunction, and autonomic phenomena (Barbero et al., 2019; Steen et al., 2025).

While MPS can affect individuals of any age, it presents unique challenges in the geriatric population. The clinical presentation is often atypical, masked by comorbidities like osteoarthritis, spinal stenosis, and diabetic neuropathy, leading to frequent misdiagnosis or underdiagnosis. This diagnostic ambiguity contributes to what can be described as a “hidden epidemic” of myofascial pain, where countless older adults suffer from a treatable condition that is often dismissed as an inevitable consequence of aging. The burden is substantial; for instance, chronic low back pain (CLBP), a condition in which MPS is a primary driver in up to 85% of cases, is the leading cause of disability worldwide (Gatchel et al., 2007; Abd-Elsayed et al., 2025). In the elderly, this translates to a vicious cycle of pain, reduced mobility, loss of independence, and increased risk of falls, frailty, and social isolation (Qaseem et al., 2017; Cheng et al., 2025).

The conventional approach to managing geriatric pain, heavily reliant on pharmacotherapy, is fraught with risk. Older adults are uniquely vulnerable to adverse drug reactions due to age-related changes in pharmacokinetics and pharmacodynamics, a high prevalence of polypharmacy, and multiple comorbidities (Cheng et al., 2025; Markovics et al., 2025). Common analgesics carry significant risks: non-steroidal anti-inflammatory drugs (NSAIDs) increase the risk of gastrointestinal bleeding and renal failure; gabapentinoids are associated with cognitive impairment, falls, and a potential increased risk of dementia (Relative Risk: 1.29); and opioids, while sometimes necessary, carry a high burden of sedation, constipation, and respiratory depression (Lipsky et al., 2024; Eghrari et al., 2025; Vamadevan et al., 2025). many medications commonly used for MPS, such as muscle relaxants, are explicitly discouraged in the elderly by the American Geriatrics Society (AGS) Beers Criteria due to their unfavorable risk–benefit profile (Oldfield et al., 2024). This pharmacological tightrope walk often results in a frustrating cycle of trial and error, with suboptimal pain relief and a high incidence of iatrogenic harm (Louw et al., 2024; Rahmatipour et al., 2025).

Prior work has established important foundations for understanding chronic pain in older adults. Miaskowski et al. (2020) proposed a comprehensive biopsychosocial model of chronic pain for older adults that integrated biological, psychological, and social dimensions, providing a foundational conceptual structure for the field. Abdulla et al. (2013) developed widely adopted evidence-based clinical practice guidelines for the management of pain in older people. More recently, Booker et al. called for adopting principles to guide pathways as the best model for geriatric pain management, acknowledging that existing evidence-based pain treatment pathways have not fully considered the unique characteristics of older adults. These seminal works have laid essential groundwork for geriatric pain management. However, several areas remain relatively underexplored. While existing frameworks have primarily focused on chronic pain as a broad category, there is an opportunity to develop disease-specific models that address the unique pathophysiology of conditions such as myofascial pain syndrome. Similarly, the rapid advances in geroscience—particularly regarding cellular senescence, mitochondrial dysfunction, and inflammaging—offer new mechanistic insights that have yet to be fully incorporated into clinical pain management frameworks.

Given the limitations of both conventional pharmacotherapy and existing broad theoretical models, it is clear that the current paradigm is failing our older adults. A fundamental shift is required, moving away from a purely biomedical, organ-centric model towards a holistic, patient-centered, and geriatrically-informed framework. Some scholars called for individualized, multimodal treatment strategies that prioritize safety, preserve function, and enhance quality of life (Markovics et al., 2025). To address these gaps, this review proposes an innovative framework built upon the convergence of transformative trends in modern medicine: geroscience, precision geriatrics, and digital therapeutics (Haythornthwaite et al., 2024). This requires viewing geriatric MPS not as an isolated musculoskeletal complaint, but as a complex geriatric syndrome rooted in the fundamental biology of aging. The geroscience hypothesis posits that by targeting the underlying mechanisms of aging, we can delay, prevent, or mitigate a wide range of age-related diseases. Applying this “geroscience lens” to MPS allows us to reframe the condition as a consequence of interacting hallmarks of aging, including sarcopenia, chronic low-grade inflammation (inflammaging), cellular senescence, and mitochondrial dysfunction.

Precision geriatrics applies the principles of precision medicine to the unique context of aging, using biomarkers, functional assessments, and multidimensional data to tailor interventions to an individual’s specific risk profile, physiological reserve, and personal goals. In MPS, this means moving beyond a generic diagnosis to phenotype the patient based on their underlying pathophysiology (e.g., sarcopenia, inflammation), functional status (e.g., frailty, mobility), and psychosocial profile.

Digital therapeutics (DTx) leverage technology to deliver evidence-based therapeutic interventions directly to patients via software programs, applications, and devices. For geriatric MPS, DTx offers an unprecedented opportunity to scale access to non-pharmacological interventions like cognitive behavioral therapy (CBT), therapeutic exercise, and patient education, overcoming barriers of mobility and access that disproportionately affect the elderly (Huang et al., 2024; Leung et al., 2024; DeBar et al., 2025; Taygar et al., 2025).

By synthesizing these concepts, this review will construct an innovative biopsychosocial framework for the management of geriatric MPS. We will first delve into the age-specific pathophysiology that makes MPS in the elderly a distinct clinical entity. We will then explore precision diagnostic approaches that combine advanced imaging with comprehensive functional assessment. Subsequently, we will critically evaluate therapeutic interventions through the lens of geriatric safety and efficacy, prioritizing non-pharmacological strategies and outlining a structured approach to precision prescribing and de-prescribing. Finally, we will discuss the integration of geriatrically-adapted psychological therapies and the role of the multidisciplinary team in orchestrating this complex care. Our goal is to provide clinicians with a comprehensive, evidence-based, and actionable roadmap to rethink their approach to myofascial pain, thereby improving the function, independence, and quality of life for this vulnerable and growing population.

## 2 Methods

### 2.1 Literature search strategy

This narrative review synthesizes evidence from multiple high-quality sources to construct an innovative biopsychosocial framework for managing myofascial pain syndrome in the elderly. We conducted a comprehensive literature search across PubMed, Web of Science, Scopus, and the Cochrane Library databases from January 2000 to December 2024. The search strategy employed combinations of Medical Subject Headings (MeSH) terms and keywords including: “myofascial pain syndrome,” “myofascial pain,” “trigger points,” “elderly,” “older adults,” “geriatric,” “aged,” “biopsychosocial,” “precision medicine,” “precision geriatrics,” “digital therapeutics,” “digital health,” “telemedicine,” “pharmacotherapy,” “de-prescribing,” “non-pharmacological treatment,” “physical therapy,” “psychological interventions,” and “multidisciplinary care.” We prioritized systematic reviews, meta-analyses, randomized controlled trials (RCTs), and high-quality observational studies published in peer-reviewed journals.

### 2.2 Selection criteria and study screening process

Studies were included if they met the following criteria: (1) focused on myofascial pain syndrome, chronic musculoskeletal pain, or related conditions in adults aged 65 years or older; (2) addressed pathophysiology, diagnosis, pharmacological treatment, non-pharmacological interventions, psychological therapies, or integrated care models; (3) published in English or Chinese; and (4) demonstrated adequate methodological quality, with a preference for studies that adhered to established reporting and methodological standards in their respective domains. Studies were excluded if they: (1) focused exclusively on pediatric or young adult populations without relevance to aging mechanisms; (2) were conference abstracts, editorials, or letters without original data or substantive review content; or (3) lacked sufficient methodological detail to assess quality.

The screening process involved two stages. In the initial screening, titles and abstracts of retrieved records were reviewed for relevance to the review’s core thematic domains. In the full-text assessment stage, potentially relevant articles were read in full to confirm eligibility. Given the multidisciplinary nature of this review, we also incorporated evidence from related fields including geriatric medicine, pain medicine, physical medicine and rehabilitation, psychology, and health technology. This cross-disciplinary approach was essential to capture the full spectrum of innovations in precision geriatrics and digital therapeutics as they apply to geriatric MPS.

As a narrative review, this work adopts a thematic synthesis approach (structuring evidence around key domains such as age-specific pathophysiology, precision diagnostics, and digital therapeutics) to allow integration of evidence across diverse disciplines and evidence types—from basic geroscience research to clinical trials to health technology assessments—which would be difficult to accommodate within a single systematic review framework. To ensure rigor, we prioritized the highest levels of evidence (systematic reviews, meta-analyses, and RCTs) where available, and transparently acknowledged when evidence was extrapolated from related populations or conditions.

### 2.3 Data synthesis and framework development

**Figure 1:**
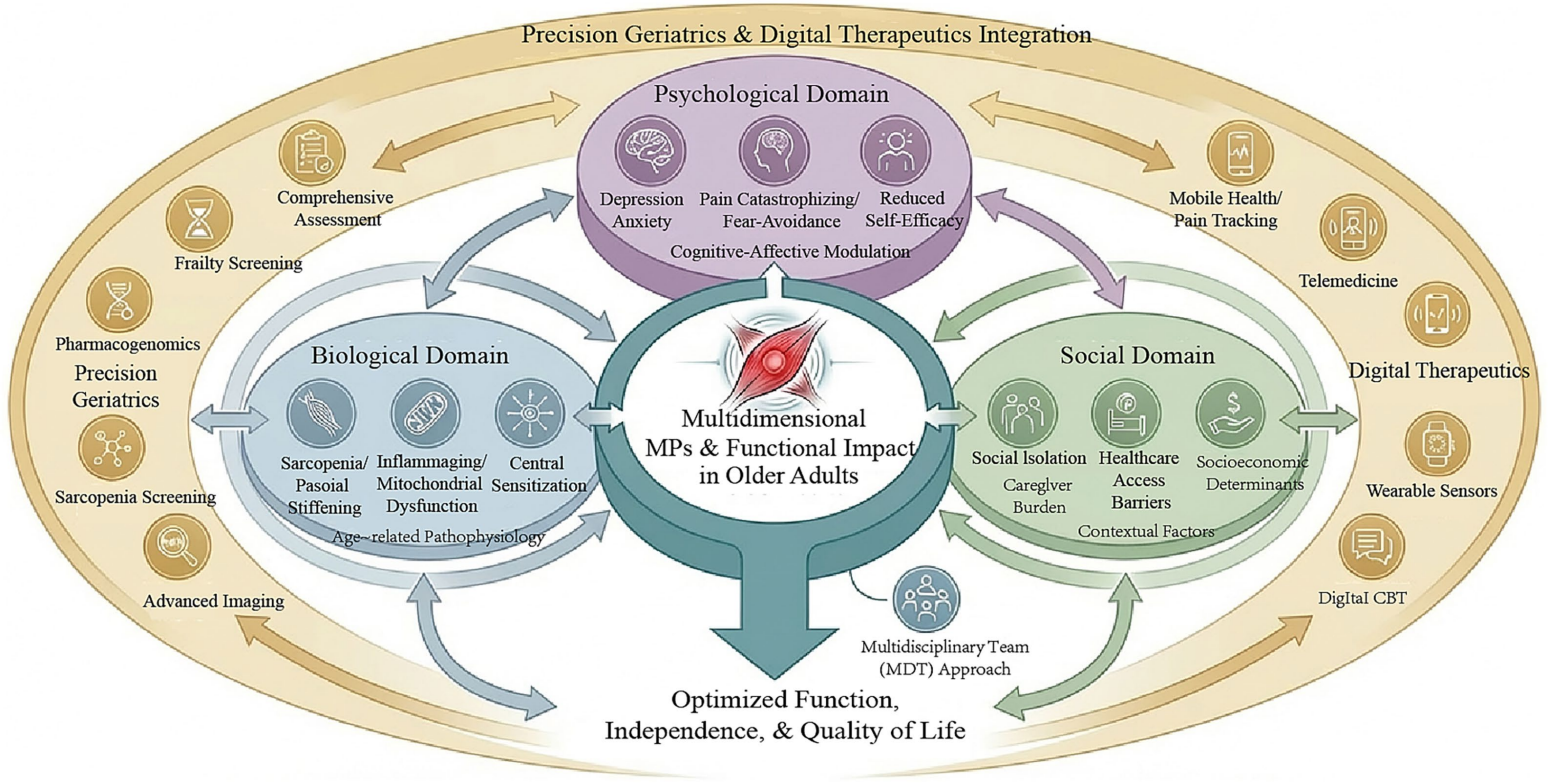
Integrated biopsychosocial framework for elderly myofascial pain syndrome. This conceptual framework illustrates the multidimensional nature of myofascial pain syndrome (MPS) in older adults, integrating biological, psychological, and social domains within precision geriatrics and digital therapeutics. The biological domain encompasses age-related pathophysiological changes including sarcopenia (progressive loss of muscle mass and function), inflammaging (chronic low-grade inflammation), fascial stiffening (reduced tissue elasticity), mitochondrial dysfunction, and central sensitization (amplified pain processing in the central nervous system). The psychological domain highlights key factors that modulate pain experience, including depression, anxiety, pain catastrophizing (exaggerated negative cognitive-affective responses to pain), fear-avoidance beliefs, and reduced self-efficacy. The social domain addresses contextual factors such as social isolation, caregiver burden, healthcare access barriers, and socioeconomic determinants. The framework emphasizes bidirectional interactions among these domains, demonstrating how biological changes can exacerbate psychological distress, which in turn amplifies pain perception and functional disability. The integration of precision geriatrics enables personalized risk stratification through comprehensive geriatric assessment, frailty screening, pharmacogenomic testing, and advanced imaging (ultrasonography, elastography). Digital therapeutics provide scalable delivery of evidence-based interventions including mobile health applications for pain tracking and self-management, telemedicine for remote consultation, wearable sensors for continuous monitoring of activity and sleep, and digital cognitive behavioral therapy platforms. The framework culminates in a patient-centered, multidisciplinary team (MDT) approach that orchestrates pharmacological and non-pharmacological interventions tailored to individual patient profiles, with the ultimate goal of optimizing function, independence, and quality of life. MPS, myofascial pain syndrome; CNS, central nervous system; MDT, multidisciplinary team; CBT, cognitive behavioral therapy.

This narrative review employed a thematic synthesis approach to organize and integrate findings. The synthesis was structured around six key domains: (1) age-specific pathophysiology, (2) precision diagnostics, (3) pharmacological management and de-prescribing, (4) non-pharmacological interventions, (5) psychological therapies, and (6) integrated multidisciplinary care. The framework emphasizes a shift from a biomedical, symptom-focused approach to a holistic, patient-centered model that addresses biological, psychological, and social dimensions of pain. It is designed to be actionable for clinicians across multiple disciplines and adaptable to diverse clinical settings (Figure 1).

## 3 The pathophysiological specificity of geriatric MPS: a geroscience-informed cascade

From a geroscience perspective, MPS can be conceptualized as a clinical manifestation of accelerated biological aging within the musculoskeletal system, where multiple hallmarks of aging converge to create a state of heightened vulnerability and impaired recovery. Understanding the pathophysiology of Myofascial Pain Syndrome (MPS) in older adults requires moving beyond the traditional model of a simple localized muscle injury. Geriatric MPS results from a complex interplay between systemic age-related physiological changes and local tissue dysfunction. This section will detail the unique cascade of events, from sarcopenia and inflammaging to central sensitization, that defines the pathophysiology of MPS in the elderly.

### 3.1 The foundation: sarcopenia, dynapenia, and fascial stiffening

The aging process is intrinsically linked with a progressive decline in musculoskeletal integrity. Sarcopenia, the age-related loss of skeletal muscle mass and strength, is a cornerstone of this decline. Beginning in the fourth decade of life, muscle mass decreases by approximately 8% per decade, accelerating after the age of 70. This is accompanied by dynapenia, the age-related loss of muscle power. This loss is not uniform; there is a preferential atrophy of type II (fast-twitch) muscle fibers, which are crucial for rapid, forceful contractions and maintaining postural stability. The remaining type I (slow-twitch) fibers are thus subjected to increased chronic mechanical load and are more susceptible to overuse and injury (Abd-Elsayed et al., 2025). In MPS, sarcopenia creates a vulnerable substrate. A weaker, smaller muscle has less physiological reserve to handle daily physical stressors, leading to micro-trauma, metabolic overload, and the initiation of the trigger point cascade. Sarcopenia is not merely a loss of muscle quantity but also involves qualitative changes in muscle composition, including increased intramuscular fat infiltration, reduced muscle fiber quality, and impaired muscle regenerative capacity (Mao et al., 2025). The intersection of sarcopenia and musculoskeletal pain creates a bidirectional relationship: sarcopenia increases vulnerability to pain through biomechanical dysfunction and reduced tissue resilience, while chronic pain promotes physical inactivity and further muscle loss, creating a vicious cycle (Grosman and Kalichman, 2025). Underpinning these changes is profound mitochondrial dysfunction. Aging muscle is characterized by a decline in mitochondrial biogenesis, reduced efficiency of the electron transport chain, and an accumulation of mitochondrial DNA mutations. This leads to a bioenergetic crisis, where the muscle cell cannot generate sufficient ATP to meet the demands of contraction and repair, contributing directly to muscle weakness, fatigue, and an impaired ability to resolve the localized energy crisis that defines a trigger point.

Compounding the effects of sarcopenia is the age-related transformation of the body’s connective tissue, particularly the fascia. Fascia is a ubiquitous, collagenous connective tissue network that envelops muscles, nerves, and organs, playing a critical role in force transmission, proprioception, and nociception (Stecco et al., 2007, 2013). With age, the fascial matrix undergoes significant changes. There is an increase in collagen cross-linking, a decrease in elastin content, and a reduction in hyaluronic acid, a key lubricant. This leads to fascial stiffening, reduced tissue compliance, and impaired gliding between fascial layers. This increased stiffness can directly contribute to pain by compressing embedded nociceptors and small nerve fibers. Stiffened fascia can alter movement patterns, leading to abnormal loading of muscles and joints, further perpetuating the cycle of micro-trauma and pain. Studies have shown that the deep fascia of the lumbar region is significantly thicker and less mobile in individuals with chronic low back pain, a condition intrinsically linked to MPS (de Williams et al., 2020).

### 3.2 The accelerator: “inflammaging” and neuroendocrine dysregulation

Superimposed on these structural changes is a systemic, pro-inflammatory state known as “inflammaging.” This refers to the chronic, low-grade, sterile inflammation that characterizes the aging process, driven by an accumulation of pro-inflammatory stimuli (e.g., senescent cells, gut dysbiosis, visceral adiposity) and a decline in the immune system’s ability to resolve inflammation (Teissier et al., 2022). A key driver of inflammaging is cellular senescence—the accumulation of non-dividing but metabolically active cells that secrete a cocktail of inflammatory mediators, known as the senescence-associated secretory phenotype (SASP). In aging muscle and fascia, senescent fibroblasts and immune cells contribute to tissue dysfunction, fibrosis, and pain persistence in older adults (Muhammad et al., 2025). Elevated levels of circulating pro-inflammatory cytokines, such as Interleukin-6 (IL-6) and Tumor Necrosis Factor-alpha (TNF-*α*), are a hallmark of inflammaging (Upadhyay et al., 2025). This systemic inflammatory milieu can lower the activation threshold of peripheral nociceptors, effectively “priming” them to fire in response to otherwise innocuous stimuli (Donovan et al., 2025; Stojanovic et al., 2025). This may explain why older adults often experience more widespread and persistent pain from a given peripheral injury and are more susceptible to the development of chronic pain states (Zhou et al., 2021; Abdelkader et al., 2025).

Age-related changes in the neuroendocrine system also play a crucial role. Dysregulation of the hypothalamic–pituitary–adrenal (HPA) axis, the body’s central stress response system, is common in older adults (Kerim et al., 2025). This can lead to blunted or altered cortisol rhythms and an impaired ability to mount an effective anti-inflammatory response to stress, further exacerbating the pro-inflammatory state of inflammaging (Knezevic et al., 2023; Nunez et al., 2025). Furthermore, deficiencies in certain hormones and nutrients are more prevalent in the elderly and have been directly linked to MPS. For instance, a high prevalence of vitamin D deficiency has been observed in patients with chronic MPS, with age and limited sun exposure being significant risk factors (Kupisz-Urbańska et al., 2021; Cirillo et al., 2022). Vitamin D is not only crucial for calcium homeostasis and bone health but also plays a vital role in muscle function and immune modulation. Its deficiency may contribute to muscle weakness (myopathy), pain, and inflammation, further fueling the MPS cycle (Agoncillo et al., 2023; Fuentes-Barría et al., 2025).

### 3.3 The amplifier: central sensitization and altered pain processing

In many older adults with chronic MPS, the pain experience is no longer solely driven by the peripheral MTrP. The constant barrage of nociceptive signals from the dysfunctional muscle tissue leads to profound neuroplastic changes in the central nervous system (CNS), a process known as central sensitization (De Schoenmacker et al., 2023). This phenomenon involves a series of molecular and cellular adaptations in the dorsal horn of the spinal cord and in higher brain centers, resulting in a state of CNS hyperexcitability (Vlodeks, 2025; Zhang M. et al., 2025; Zhang S. et al., 2025).

At the spinal level, this includes the phosphorylation of NMDA receptors, increased expression of substance P, and the activation of glial cells (microglia and astrocytes), which release their own pro-inflammatory mediators, creating a neuroinflammatory state within the spinal cord (Zhonghua et al., 2025). The consequence of this is two fold: hyperalgesia, an exaggerated pain response to a noxious stimulus, and allodynia, the perception of pain in response to a normally non-painful stimulus (such as light touch) (Jensen and Finnerup, 2014). This is why patients with chronic MPS often report that their pain is spreading beyond the original site and that even the light pressure of clothing can be uncomfortable (Volcheck et al., 2023).

These changes are not confined to the spinal cord. Central sensitization involves a rewiring of pain processing circuits in the brain. Brain imaging studies in chronic pain patients have revealed altered activity and connectivity in key regions of the “pain matrix,” including the prefrontal cortex, anterior cingulate cortex (ACC), insula, and thalamus (Nara et al., 2025). There is often a shift from acute nociceptive processing to circuits involved in emotion and salience, explaining why chronic pain becomes so emotionally distressing and all-consuming (Zhang et al., 2022).

Psychological factors, which are highly prevalent in older adults with chronic pain, act as powerful top-down modulators of this centralized pain state (Zhang M. et al., 2025; Zhang S. et al., 2025). Pain catastrophizing (a negative cognitive-affective response to pain), anxiety, and depression can directly amplify the activity in these emotional brain circuits, further enhancing the perception of pain and creating a vicious, self-perpetuating cycle (Haythornthwaite et al., 2024). This is the neurobiological basis of the biopsychosocial model of pain: the pain experience is an emergent property of the dynamic interaction between peripheral nociception, central processing, and psychological state (Armstrong et al., 2024).

**Figure 2:**
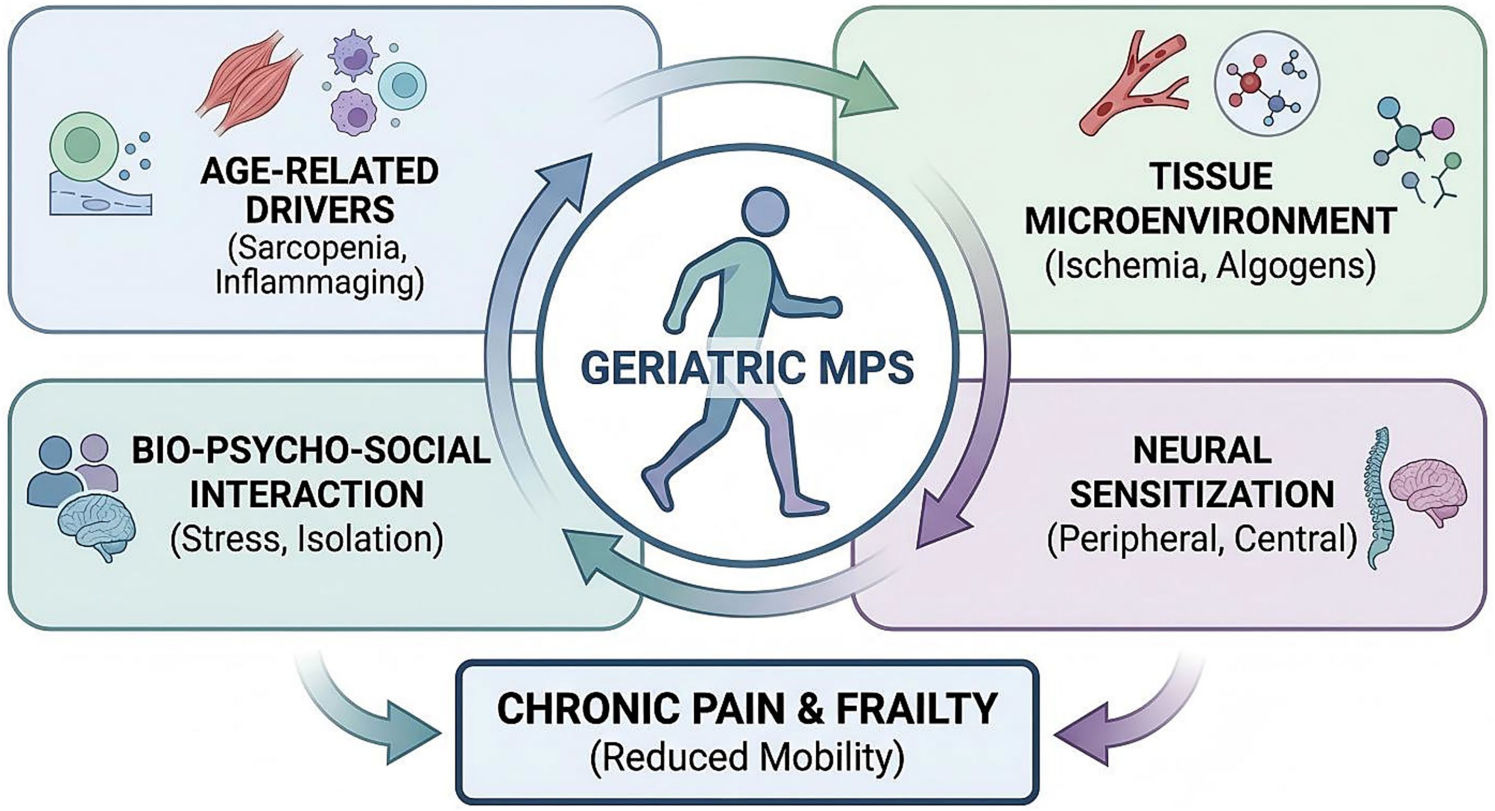
Age-specific pathophysiological cascade in geriatric myofascial pain syndrome. This schematic diagram depicts the complex, interconnected pathophysiological mechanisms that underlie myofascial pain syndrome in the elderly, emphasizing how age-related changes create a self-perpetuating cycle of pain and dysfunction. The cascade begins with primary age-related changes: (1) Sarcopenia, characterized by loss of type II muscle fibers, reduced muscle strength, and impaired regenerative capacity; (2) Inflammaging, a state of chronic systemic inflammation driven by elevated pro-inflammatory cytokines (IL-6, TNF-*α*, IL-1β) and cellular senescence; (3) Fascial changes, including increased collagen cross-linking, reduced hyaluronic acid content, and decreased tissue hydration leading to stiffness; and (4) Mitochondrial dysfunction, resulting in impaired energy metabolism and increased oxidative stress. These primary changes converge to produce local tissue pathology at the trigger point level, including chronic muscle contraction, microcirculatory insufficiency (ischemia), accumulation of algogenic substances (bradykinin, substance P, glutamate), and neurogenic inflammation. The local pathology triggers peripheral sensitization, characterized by reduced nociceptor thresholds and spontaneous activity in primary afferent neurons. Persistent nociceptive input from peripheral tissues drives maladaptive neuroplasticity in the spinal cord and brain, resulting in central sensitization—a state of hyperexcitability of central pain pathways that amplifies pain signals and expands receptive fields. Central sensitization is further modulated by descending pain modulation pathways, which are often impaired in the elderly, leading to reduced endogenous pain inhibition. Psychological factors (depression, anxiety, catastrophizing) and social factors (isolation, stress) interact with central sensitization through shared neurobiological substrates (e.g., limbic system, prefrontal cortex), creating a vicious cycle. The end result is chronic, widespread pain with associated functional impairments including reduced mobility, fear-avoidance behavior, deconditioning, and progressive frailty. Arrows indicate causal relationships and feedback loops. This framework underscores the need for multimodal interventions that target multiple nodes in the cascade rather than a single mechanism. IL, interleukin; TNF-α, tumor necrosis factor-alpha; ATP, adenosine triphosphate; ROS, reactive oxygen species.

In summary, MPS in the elderly is not simply a muscle problem; it is a systemic condition rooted in the biology of aging. It begins with the vulnerable substrate of sarcopenic muscle, compromised by mitochondrial energy deficits and encased in stiffened fascia, is accelerated by the pro-inflammatory milieu of inflammaging, and is amplified and sustained by the neuroplastic changes of central sensitization. Acknowledging this complex, multi-level pathophysiology is the essential first step toward developing a more effective, holistic, and geriatrically-informed management approach that targets the condition at each of these levels. Figure 2 illustrates this complex, multi-level pathophysiological cascade that underlies MPS in the elderly.

## 4 Diagnostic innovations: balancing precision with safety in geriatric assessment

The diagnosis of MPS has traditionally been a clinical skill, relying on the practitioner’s ability to identify a taut band and a hypersensitive MTrP through manual palpation that reproduces the patient’s characteristic pain (Barbero et al., 2019). While this clinical examination remains the cornerstone of diagnosis, its inherent subjectivity has led to limited inter-rater reliability, posing a significant challenge for both clinical practice and research. In the geriatric population, this challenge is magnified. Communication difficulties due to hearing or cognitive impairment, altered pain perception, and the presence of multiple overlapping pain sources can make a purely palpation-based diagnosis unreliable. A more objective, precise, and multidimensional diagnostic approach is therefore imperative (Duarte et al., 2021).

### 4.1 Visualizing the pathology: musculoskeletal ultrasonography and elastography

Musculoskeletal ultrasonography has emerged as a powerful, non-invasive, and cost-effective tool for visualizing MTrPs and guiding interventions. Unlike MRI or CT, ultrasound provides a dynamic, real-time assessment of soft tissues without exposing the patient to radiation (Chen et al., 2024). MTrPs typically appear on ultrasound as focal, hypoechoic (darker) regions within the muscle, often with a disorganized or lost normal fibrillar pattern (Wu et al., 2024). Ultrasound can also identify associated abnormalities such as fascial thickening, fluid collections, or inflammation in adjacent bursae, helping to differentiate MPS from other conditions (Cao et al., 2025).

Elastography is an advanced ultrasound technique that takes this visualization a step further by measuring tissue stiffness. The underlying principle is that the sustained contraction and localized edema within an MTrP make it significantly stiffer than the surrounding healthy, pliable muscle tissue (Tsai et al., 2024). Several forms of elastography exist, but Shear Wave Elastography (SWE) is particularly promising. SWE uses an acoustic radiation force impulse to generate a small shear wave that propagates through the tissue. The speed of this wave, which is directly related to tissue stiffness, is measured and quantified in kilopascals (kPa) or meters per second (m/s) (Tsai et al., 2024; López-Redondo et al., 2025). This provides an objective, quantitative biomarker for identifying MTrPs and, potentially, for monitoring treatment response. A recent study showed that ultrasound measurements of MTrP size and elasticity can effectively differentiate their characteristics in lower back muscles, supporting its use in clinical assessment (Wu et al., 2022; Lei et al., 2025). For older adults, the safety, accessibility, and affordability of ultrasound and elastography make them ideal tools for enhancing diagnostic precision (Nakagawa et al., 2025).

### 4.2 Beyond the trigger point: functional and multidimensional assessment

A diagnosis of MPS in an older adult should not end with the identification of a trigger point. A comprehensive geriatric assessment must evaluate the broader impact of pain on the patient’s function, well-being, and overall health. This requires a multidimensional approach that extends far beyond the localized pain (Koren et al., 2022).

Functional Mobility Assessment: Standardized, performance-based tests are essential to quantify the impact of pain on mobility. Key measures include:

- Timed Up and Go (TUG) Test: Measures the time it takes for a person to rise from a chair, walk 3 meters, turn around, and sit down again. It is a quick and reliable indicator of balance, gait speed, and fall risk (Choo et al., 2021).
- 6-Minute Walk Test (6MWT): Assesses functional exercise capacity by measuring the distance a patient can walk in 6 min. It reflects endurance and cardiovascular fitness (Agarwala and Salzman, 2020).
- Gait Speed: Often considered the “sixth vital sign” in geriatrics, gait speed is a powerful predictor of future disability, hospitalization, and mortality. A slow gait speed (<1.0 m/s) is a red flag for functional decline (de Souza et al., 2025).

Frailty Assessment: Frailty is a state of increased vulnerability to stressors due to age-related declines in multiple physiological systems. It is a critical prognostic indicator and should be systematically assessed. Validated tools include the Fried Frailty Phenotype (which assesses weight loss, exhaustion, low physical activity, slowness, and weakness) and the Clinical Frailty Scale, a simpler, judgment-based tool (Küçük et al., 2025).

Psychosocial Screening: As discussed, psychological factors are potent amplifiers of chronic pain. A comprehensive assessment must therefore include screening for:

- Depression and Anxiety: Using validated scales like the Geriatric Depression Scale (GDS) and the Geriatric Anxiety Inventory (GAI).
- Pain Catastrophizing: The Pain Catastrophizing Scale (PCS) measures ruminative, magnifying, and helpless thoughts about pain.
- Fear-Avoidance Beliefs: The Fear-Avoidance Beliefs Questionnaire (FABQ) assesses how fear of pain may be limiting physical activity.

Nutritional and Metabolic Assessment: Given the high prevalence of nutritional deficiencies in the elderly, screening for conditions like vitamin D deficiency is crucial. Assessing for metabolic syndrome is also important, as it is linked to chronic inflammation (Farid et al., 2025).

By integrating these objective imaging and functional measures with a thorough clinical and psychosocial evaluation, clinicians can construct a multidimensional profile of the patient. This “precision diagnostics” approach allows for a more accurate diagnosis, better risk stratification, and the development of a truly personalized treatment plan that addresses all contributing factors—moving far beyond simply identifying a sore spot in a muscle.

## 5 Therapeutic framework: a geroscience-informed multimodal approach

Building on our geroscience-informed understanding of geriatric MPS, the therapeutic framework must be equally comprehensive, targeting the condition at multiple levels: mitigating systemic drivers like inflammaging, restoring local tissue health, recalibrating centralized pain circuits, and empowering the patient with self-management skills. This section outlines a multimodal, integrated approach that prioritizes non-pharmacological interventions, employs precision prescribing and deprescribing, and leverages digital therapeutics to enhance access and engagement.

### 5.1 Pharmacotherapy: precision prescribing and de-prescribing in the elderly

Pharmacological management of MPS in older adults is a delicate balancing act, a clinical tightrope walk between providing symptomatic relief and avoiding iatrogenic harm. The elderly are at a significantly higher risk for adverse drug reactions (ADRs), drug–drug interactions, and medication-related hospitalizations due to a confluence of factors: age-related changes in pharmacokinetics (absorption, distribution, metabolism, excretion) and pharmacodynamics (drug-receptor interactions), the high prevalence of polypharmacy, and the presence of multiple comorbidities (Rodriguez-Espeso et al., 2025). The traditional, often empirical, approach of prescribing analgesics must give way to a more thoughtful, evidence-based strategy that prioritizes safety, individualizes treatment, and actively embraces de-prescribing—the systematic process of tapering or stopping medications that may no longer be beneficial or may be causing harm (Liacos et al., 2020; American Geriatrics Society Beers Criteria® Alternatives Panel, 2023).

### 5.2 A critical re-evaluation: risk–benefit of common analgesics

Many medications commonly prescribed for chronic pain have a questionable or unfavorable risk–benefit profile in the geriatric population.

Non-Steroidal Anti-Inflammatory Drugs (NSAIDs): While effective for acute inflammatory pain, the chronic use of NSAIDs (e.g., ibuprofen, naproxen) is strongly discouraged in the elderly. They carry significant risks of gastrointestinal bleeding, peptic ulcer disease, acute renal failure, and exacerbation of hypertension and heart failure (Hopkins et al., 2025). The AGS Beers Criteria recommend avoiding chronic use unless other alternatives are not effective and the patient can take a gastroprotective agent (American Geriatrics Society Beers Criteria® Alternatives Panel, 2023; American Geriatrics Society Beers Criteria® Alternatives Panel, Steinman MA, 2025).

Gabapentinoids (Gabapentin and Pregabalin): These drugs are widely prescribed for neuropathic pain, but their efficacy in nociceptive or mixed pain states like MPS is limited (Mayoral et al., 2024). More importantly, they are associated with a high burden of side effects in the elderly, including dizziness, somnolence, peripheral edema, and cognitive impairment, all of which significantly increase the risk of falls (Gahr, 2025). A recent study even linked gabapentin prescription in older adults with chronic low back pain to an increased risk of developing dementia (RR: 1.29) (Eghrari et al., 2025). Their use should be highly selective and initiated at very low doses.

Skeletal Muscle Relaxants: Medications like cyclobenzaprine have very limited evidence of efficacy beyond their sedative effects (Oldfield et al., 2024). They are potent anticholinergics, leading to a high risk of confusion, constipation, dry mouth, and falls (Coli et al., 2024). They are strongly discouraged by the AGS Beers Criteria and should generally be avoided in older adults (American Geriatrics Society Beers Criteria® Alternatives Panel, 2023).

Tricyclic Antidepressants (TCAs): Amitriptyline, once a mainstay for chronic pain, also has a high anticholinergic burden and carries risks of cardiac arrhythmias and orthostatic hypotension. Current evidence suggests that newer agents, such as duloxetine, show more consistent efficacy with moderate-to-high certainty (Birkinshaw et al., 2023).

Serotonin-Norepinephrine Reuptake Inhibitors (SNRIs): Serotonin-Norepinephrine Reuptake Inhibitors (SNRIs) have showed efficacy for chronic musculoskeletal pain, including back pain and fibromyalgia, with moderate certainty evidence. Duloxetine at 60 mg has been shown to significantly reduce pain and improve quality of life in chronic nonspecific low back pain, with generally tolerable side effects, though higher doses may increase adverse event (Skljarevski et al., 2010; Birkinshaw et al., 2023).

### 5.3 The imperative of de-prescribing: a structured framework

Given the risks associated with polypharmacy, de-prescribing is not just an option but a critical component of geriatric MPS management. It is a patient-centered process that requires a systematic approach.

1. Comprehensive Medication Review: The first step is to create a complete list of all medications, including prescriptions, over-the-counter drugs, and supplements.
2. Identify High-Risk Medications: Using tools like the AGS Beers Criteria and the STOPP/START criteria (Screening Tool of Older Persons’ Prescriptions/Screening Tool to Alert to Right Treatment), identify potentially inappropriate medications.
3. Assess Each Medication: For each drug, assess the risk–benefit ratio. Is the indication still present? Is the medication providing a clear benefit? Are there adverse effects? Could the therapeutic objective be achieved with a safer alternative?
4. Prioritize for Discontinuation: Prioritize high-risk, low-benefit medications for discontinuation. Muscle relaxants, benzodiazepines, and long-term NSAIDs are often prime candidates.
5. Shared Decision-Making: Discuss the rationale for de-prescribing with the patient and their caregivers. Address their fears and expectations and create a collaborative plan.
6. Gradual Tapering and Monitoring: Use a slow, gradual tapering protocol to avoid withdrawal symptoms or rebound pain. Monitor the patient closely during and after the taper.

**Figure 3:**
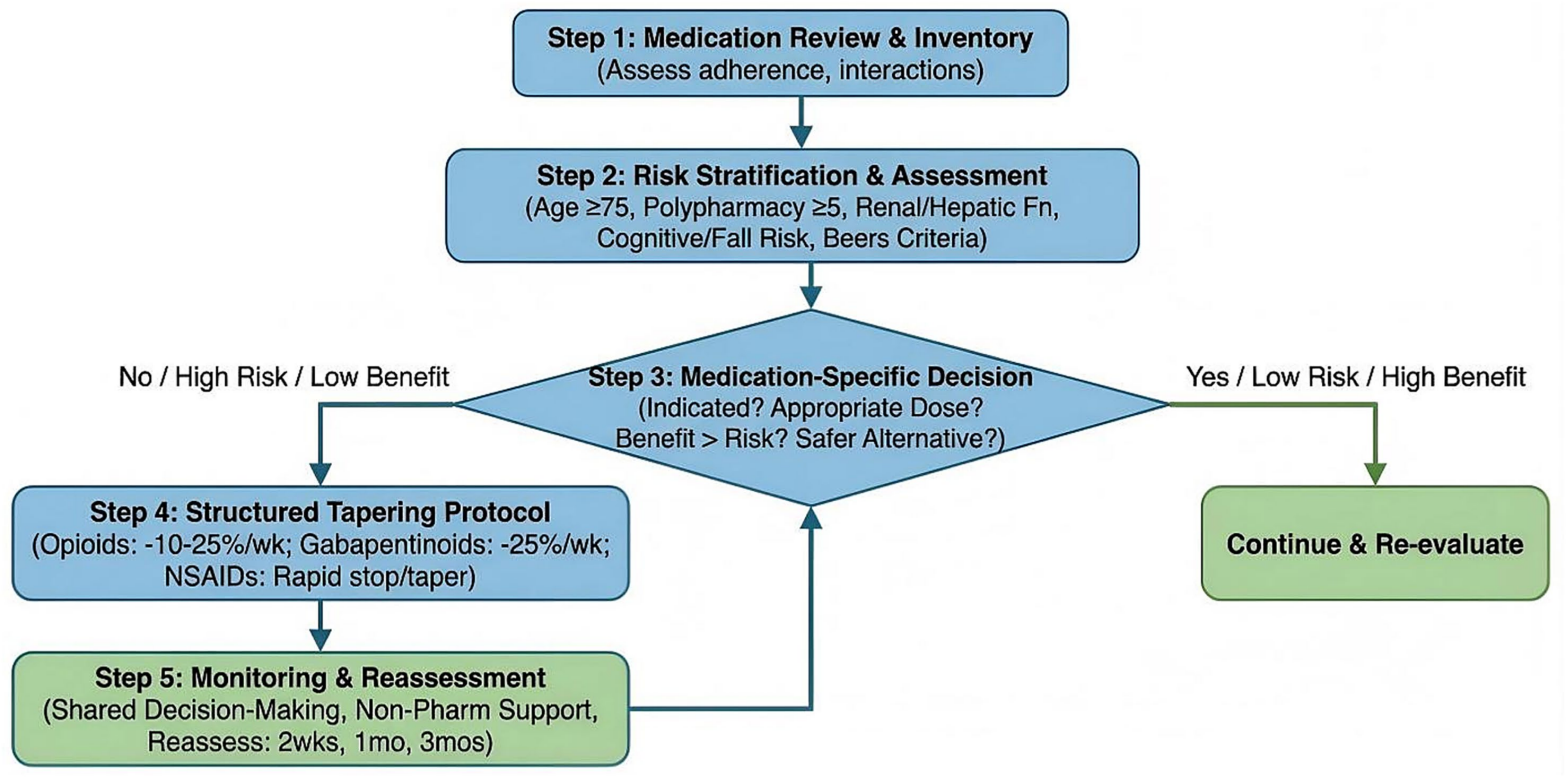
Structured de-prescribing framework for analgesics in elderly patients with myofascial pain syndrome. This clinical decision flowchart provides a systematic, stepwise approach to de-prescribing—the process of tapering or discontinuing medications that may no longer be beneficial or may be causing harm—in older adults with myofascial pain syndrome. The framework is designed to enhance medication safety, reduce polypharmacy, and improve the risk–benefit balance of analgesic therapy in the geriatric population. The process begins with a comprehensive medication review, during which the clinician inventories all current medications (prescription, over-the-counter, supplements), assesses adherence, and identifies potential drug–drug interactions and adverse effects. The next step is risk stratification, which evaluates patient-specific factors including age (≥75 years as higher risk), number of medications (≥5 as polypharmacy threshold), renal and hepatic function (estimated glomerular filtration rate, liver enzymes), cognitive status (using tools such as the Montreal Cognitive Assessment), fall history, and presence of high-risk medications as defined by the American Geriatrics Society Beers Criteria (e.g., NSAIDs, benzodiazepines, anticholinergics, gabapentinoids). For each medication, the clinician then applies a decision algorithm: (1) Is there a clear indication for continued use? (2) Is the current dose appropriate for the patient’s age and renal/hepatic function? (3) Are there documented benefits (e.g., pain reduction, improved function)? (4) Are there current or potential adverse effects? (5) Is there a safer alternative (non-pharmacological or alternative medication)? Medications flagged as high-risk or low-benefit proceed to a structured tapering protocol. Tapering schedules are medication-specific: for example, opioids are reduced by 10–25% every 1–2 weeks with close monitoring for withdrawal symptoms; gabapentinoids are tapered by 25% per week; and NSAIDs can often be stopped abruptly if no contraindications exist, with gastroprotection maintained during the taper if needed. Throughout the de-prescribing process, the framework emphasizes shared decision-making with the patient and family, patient education about the rationale for de-prescribing, close monitoring for pain flares or withdrawal symptoms (with contingency plans for temporary dose adjustments), and simultaneous optimization of non-pharmacological interventions (physical therapy, psychological support, lifestyle modifications) to maintain pain control. The framework includes decision points for reassessment at 2 weeks, 1 month, and 3 months post-taper, with criteria for success (stable or improved pain and function, reduced adverse effects) and failure (unacceptable pain increase, functional decline), guiding whether to continue, pause, or reverse the taper. This evidence-based, patient-centered approach aims to reduce medication burden while maintaining or improving quality of life. NSAIDs, non-steroidal anti-inflammatory drugs; AGS, American Geriatrics Society; eGFR, estimated glomerular filtration rate.

We propose a structured, stepwise de-prescribing framework (Figure 3) that prioritizes safety while maintaining pain control.

### 5.4 The future of analgesia: novel targets and precision approaches

The future of pharmacotherapy for MPS lies in developing more targeted agents with better safety profiles. Selective Sodium Channel Blockers: A highly promising frontier is the development of selective inhibitors of the NaV1.8 sodium channel. This channel is preferentially expressed in peripheral nociceptive (pain-sensing) neurons and plays a crucial role in signaling pain. By selectively blocking NaV1.8, it is possible to achieve potent analgesia without the central nervous system side effects (like sedation, confusion, or addiction) associated with opioids and other centrally-acting drugs (Osteen et al., 2025). Suzetrigine (VX-548) is a first-in-class NaV1.8 inhibitor that has shown significant analgesic efficacy in Phase III trials for acute pain, and was approved by the FDA in January 2025 as the first non-opioid selective NaV1.8 inhibitor (Bertoch et al., 2025; McCoun et al., 2025). While long-term data in chronic pain are still needed, these agents could offer a transformative, safer option for geriatric MPS (Dorta et al., 2024).

Other Novel Targets: Other emerging targets include Transient Receptor Potential A1 (TRPA1) antagonists (Broad et al., 2025), which have shown promise in early clinical trials for chronic pain states (Lagarde et al., 2024), and Nerve Growth Factor (NGF) monoclonal antibodies, although their development has been hampered by safety concerns, most notably the risk of rapidly progressive osteoarthritis observed in clinical trials (Hochberg, 2015).

### 5.5 Senolytic therapies: a geroscience-targeted frontier

A pharmacological frontier with particular relevance to geriatric MPS is senolytic therapy—agents designed to selectively eliminate senescent cells that accumulate with aging and drive chronic inflammation through the senescence-associated secretory phenotype (SASP). Given the central role of cellular senescence and inflammaging in the pathophysiology of geriatric MPS as outlined in this review, senolytics represent a mechanistically targeted approach. Preclinical studies have demonstrated that senescent cells accumulate in musculoskeletal tissues and contribute to chronic pain, tissue fibrosis, and impaired regeneration (Falvino et al., 2024). The combination of dasatinib and quercetin (D + Q) was the first senolytic regimen tested in human clinical trials, with initial results demonstrating feasibility and preliminary efficacy in reducing senescent cell burden (Hickson et al., 2019). More recently, senolytic approaches have been investigated specifically for musculoskeletal pain conditions. A 2025 study reported senolytic treatment strategies for low back pain, with ongoing clinical trials evaluating quercetin, curcumin, and UBX0101 for intervertebral disc degeneration and associated pain (Mannarino et al., 2025). A comprehensive review of senolytic drugs in orthopedic diseases further highlighted their potential for conditions driven by cellular senescence, including osteoarthritis and degenerative musculoskeletal disorders (Zhang M. et al., 2025; Zhang S. et al., 2025). While these agents remain in early clinical development and no trials have specifically targeted MPS, the mechanistic rationale is compelling: by reducing the senescent cell burden in aging muscle and fascia, senolytics could potentially address a root cause of the inflammaging-driven pain amplification cycle that characterizes geriatric MPS. Significant challenges remain, however, including establishing optimal dosing regimens, long-term safety profiles, and identifying biomarkers to select patients most likely to benefit.

### 5.6 Rethinking injection therapies: ultrasound-guided, low-risk approaches

Injection therapies remain a valuable tool for targeting localized MTrPs, but the approach must be refined for the elderly.

Ultrasound-Guided Trigger Point Injections (TPIs): Ultrasound guidance should now be considered the standard of care for TPIs. It enhances both safety (by visualizing and avoiding nerves and blood vessels) and efficacy (by ensuring the injectate is delivered precisely to the MTrP) (Thu, 2022; Wu et al., 2024). The most common and best-supported injectate is a local anesthetic (e.g., lidocaine or bupivacaine). The use of corticosteroids in TPIs is controversial and generally not recommended due to the risk of local muscle atrophy. Dry needling, the insertion of a needle without any injectate, is also an effective option that avoids any drug-related side effects (Anwar et al., 2024). The efficacy of Botulinum toxin A for MPS remains a subject of debate. While some earlier studies found it to be no more effective than placebo or local anesthetic (Soares et al., 2014), recent updated reviews highlight its versatility and promising role in providing significant pain relief and functional improvement for MPS (Rahmatipour et al., 2025).

Regenerative Medicine Approaches: Platelet-rich plasma is an emerging regenerative approach that has been proposed for MPS management. Limited evidence from small studies suggests potential benefits. A retrospective case series reported pain reduction compared to lidocaine injection, with the proposed mechanism involving growth factor-mediated tissue repair and anti-inflammatory effects (Ai et al., 2025). However, critical evaluation reveals substantial concerns that preclude definitive recommendations, particularly for older adults. Evidence Quality Concerns: The evidence base for PRP in MPS is notably weak. Current systematic reviews highlight a scarcity of high-quality randomized controlled trials specifically investigating myofascial pain syndrome, particularly in older adult populations. The theoretical mechanism of PRP—promoting tissue regeneration through growth factor release—is more compelling for structural pathology with tissue damage (e.g., tendinopathy, osteoarthritis, ligament injuries) than for MPS, which is fundamentally a functional neuromuscular disorder without significant structural tissue damage (Yi et al., 2025). The absence of established predictive biomarkers for PRP response hinders clinical interpretation and the implementation of personalized treatment strategies (Haizhou et al., 2025). Economic and Equity Considerations: PRP costs $500–2,500 per treatment session in the United States, typically requiring 2–3 sessions for a full course, yet remains largely uninsured due to insufficient evidence. Total out-of-pocket costs of $1,000–7,500 create significant access barriers and raise ethical concerns about promoting expensive interventions with uncertain benefits to vulnerable older adults on fixed incomes (Tiao et al., 2024). Cost-effectiveness analyses are notably absent from the literature—a critical gap given limited healthcare resources (Raeissadat et al., 2023). Based on current evidence, PRP should be considered experimental for geriatric MPS and cannot be recommended as standard care. It may be reasonable to consider PRP in highly selected, refractory cases who have failed multiple evidence-based treatments, but only within research protocols with rigorous informed consent, standardized protocols, and systematic outcome tracking (Ai et al., 2025).

## 6 Non-pharmacological interventions: the first line of geriatric MPS management

In the management of MPS in older adults, a fundamental paradigm shift is needed, elevating non-pharmacological interventions from their traditional role as “adjunctive” therapies to the status of first-line treatments. This shift is driven by a confluence of factors: the compelling evidence of their efficacy, their vastly superior safety profiles compared to pharmacotherapy, and the ethical and clinical imperative to minimize the risks associated with polypharmacy in the elderly (Vaegter et al., 2024; Briggs et al., 2025). The goal of non-pharmacological management is not just to reduce pain, but to restore function, improve mobility, and enhance self-efficacy, thereby breaking the vicious cycle of pain and disability.

### 6.1 Movement as medicine: evidence-based therapeutic exercise

Movement and exercise are foundational to the management of chronic musculoskeletal pain. The principle of “motion is lotion” is particularly apt for MPS, where inactivity leads to muscle shortening, fascial stiffness, and increased pain. A network meta-analysis of exercise interventions for chronic low back pain found that several specific modalities were superior to usual care in improving both pain and function (Li et al., 2023).

Tai Chi: This ancient Chinese practice combines slow, flowing movements, deep breathing, and meditation. It is particularly well-suited for older adults as it is low-impact and has been shown to improve not only pain and function but also balance, flexibility, and proprioception, thereby directly addressing key risk factors for falls. Its meditative component also helps to reduce stress and pain catastrophizing (Qin et al., 2025).

Yoga: Like Tai Chi, yoga integrates physical postures (asanas), breathing techniques (pranayama), and meditation. It has been shown to be effective for chronic low back pain and other musculoskeletal conditions (Xia et al., 2025). For older adults, yoga practice must be adapted, with a focus on gentle stretches, chair-based modifications, and an avoidance of extreme ranges of motion (Silva A. G. et al., 2025; Silva M. T. et al., 2025).

Aquatic Exercise: Exercising in a warm water pool is an excellent option for frail older adults or those with severe pain or arthritis. The buoyancy of the water reduces joint loading and impact, allowing for a greater range of motion and strengthening exercises that might be too painful to perform on land (Wang et al., 2023).

When prescribing exercise for older adults, clinicians should adhere to the FITT-VP principles (Frequency, Intensity, Time, Type, Volume, and Progression), starting at a low intensity and progressing gradually as tolerated to avoid injury and build confidence (Cheng et al., 2025).

### 6.2 Hands-on approaches: optimized manual therapy and physical modalities

Manual therapy and physical modalities can provide significant symptomatic relief and help engagement in active exercise programs.

Manual Therapy: Techniques such as massage, myofascial release, and trigger point pressure release have been shown to be effective for MPS, though the quality of evidence varies (He et al., 2023). One particularly promising technique for the elderly is Strain-Counterstrain (SCS). SCS is a gentle, indirect technique where the affected muscle is passively moved into a position of maximal comfort, which is held for about 90 s. This is thought to reset the dysfunctional neuromuscular feedback loops that maintain the trigger point. An RCT showed that SCS combined with exercise significantly reduced pain severity and improved lumbar range of motion and functional capacity in patients with chronic low back pain and MPS compared to exercise alone (He et al., 2023; Koura et al., 2025). Its gentle nature makes it well-tolerated even in frail patients.

Physical Modalities: A systematic review found that several physical modalities can improve pain, range of motion, and quality of life in MPS patients with no reported side effects (He et al., 2023). These include:

- Transcutaneous Electrical Nerve Stimulation (TENS): A non-invasive technique where a low-voltage electrical current is delivered through electrodes on the skin to stimulate sensory nerves and provide pain relief via the gate control theory of pain (Steen et al., 2025).
- Extracorporeal Shockwave Therapy (ESWT): This modality uses acoustic waves to stimulate tissue healing and reduce pain. A randomized sham-controlled trial found that high-energy focused ESWT was safe and effective for chronic lumbar facet pain, providing long-term relief (Gerhart et al., 2023).
- Laser Therapy: Laser therapy and extracorporeal shockwave therapy were found to be more effective than no treatment in reducing pain intensity and improving pressure pain threshold in MPS patients (Liu et al., 2024).

### 6.3 The future of rehabilitation: neuromodulation techniques

Neuromodulation represents an exciting frontier in pain rehabilitation, using electrical or magnetic energy to modulate the activity of the nervous system.

Repetitive Peripheral Magnetic Stimulation (rPMS): This non-invasive technique uses a powerful magnet to generate a focused magnetic field that can penetrate deep into tissues and induce electrical currents in nerves. This can be used to directly stimulate muscles, modulate neuromuscular activation, and enhance endogenous pain control mechanisms. A systematic review and meta-analysis showed that rPMS significantly reduces pain intensity and improves function in patients with chronic musculoskeletal pain, especially chronic low back pain (Pan et al., 2025). Combining ultrasound-guided injections with rPMS offers a promising, minimally invasive multimodal strategy (Thu, 2022).

Restorative Neurostimulation Therapy: This is a more invasive approach that involves the implantation of electrodes to directly stimulate the multifidus muscle, a key spinal stabilizer that is often atrophied and dysfunctional in chronic low back pain. By restoring the activation of this muscle, the therapy aims to improve spinal stability and reduce pain. A major RCT showed significant and durable improvements in pain, disability, and quality of life in patients with chronic mechanical low back pain refractory to other treatments (Schwab et al., 2025). While currently indicated for a specific subset of patients, it highlights the trend towards restorative, function-focused interventions.

By prioritizing this diverse array of non-pharmacological interventions, clinicians can create a robust, multimodal treatment plan that is not only effective but also aligns with the geriatric principle of “first, do no harm.”

## 7 Psychological interventions: innovations adapted for geriatric cognitive and affective needs

Chronic pain is never just a physical sensation; it is a complex and distressing biopsychosocial experience. The emotional suffering, or the affective dimension of pain, is often more disabling than the nociceptive input itself. This is particularly true in older adults, where chronic pain is frequently comorbid with depression, anxiety, social isolation, and a loss of personal identity (Gatchel et al., 2007; Zhang M. et al., 2025; Zhang S. et al., 2025). Therefore, psychological interventions are not an optional add-on but a central component of effective geriatric MPS management. The goal of these therapies is to change the relationship with pain—to reduce its emotional impact, improve coping skills, and re-engage with a meaningful life despite the persistence of pain.

### 7.1 The gold standard and its evolution: CBT and ACT

Cognitive Behavioral Therapy (CBT): CBT is the most widely studied and well-established psychological intervention for chronic pain (Turk et al., 2011). The core principle of CBT is that our thoughts, feelings, and behaviors are interconnected. CBT for pain helps patients to: (1) Identify and Challenge Maladaptive Thoughts: Patients learn to recognize and reframe negative thought patterns like pain catastrophizing (e.g., “This pain is a disaster and it will never get better”). (2) Modify Maladaptive Behaviors: Patients are guided to gradually overcome fear-avoidance behaviors and re-engage in valued activities through techniques like activity pacing and graded exposure. (3) Learn Active Coping Skills: This includes relaxation techniques, problem-solving, and communication skills.

For older adults, standard CBT protocols often require adaptation (Kiosses et al., 2017). Geriatrically-adapted CBT involves using simplified content, shorter sessions, larger font sizes in written materials, and integrating age-specific concerns such as grief, loss, and legacy (Haythornthwaite et al., 2024).

Acceptance and Commitment Therapy (ACT): ACT is a newer, “third-wave” cognitive-behavioral therapy that has shown significant promise for chronic pain (Ma et al., 2023). Rather than trying to change or eliminate pain, ACT focuses on increasing psychological flexibility (Moens et al., 2022). This is the ability to accept uncomfortable thoughts and feelings (like pain) while still taking action that is consistent with one’s personal values (Hughes et al., 2017; Ye et al., 2024). The core processes of ACT include: (1) Acceptance: Making room for painful sensations, thoughts, and emotions without struggling with them. (2) Cognitive Defusion: Learning to see thoughts as just thoughts, rather than objective truths or commands. (3) Contact with the Present Moment: Paying attention to the here and now (mindfulness). (4) Values Clarification: Identifying what is most important in one’s life (e.g., family, creativity, community). (5) Committed Action: Taking concrete steps toward living a life aligned with those values, even in the presence of pain. (6) Self-as-Context: Fostering an observing sense of self that is distinct from one’s thoughts and feelings.

For older adults, ACT can be particularly powerful as it resonates with themes of legacy and making the most of one’s remaining time. Age-specific ACT protocols may use simplified metaphors and integrate the therapy with life review exercises (Gerhart et al., 2023).

### 7.2 Scaling access through technology: digital psychological therapy platforms

One of the greatest barriers to psychological care for older adults is access—due to mobility issues, transportation difficulties, or a shortage of trained therapists (Tao et al., 2023). Digital therapeutics (DTx) offer a scalable and effective solution to this problem. A growing body of high-quality clinical evidence supports the efficacy of digital psychological interventions for chronic pain. Telehealth and online CBT-based treatments have been shown to significantly improve pain interference and physical function in patients with high-impact chronic pain, with effects sustained at 6-month follow-up (DeBar et al., 2025). In the domain of fibromyalgia—a condition sharing central sensitization mechanisms with chronic MPS—self-guided digital behavioral therapy significantly reduced pain severity and improved global function compared to active control in a phase 3, multicenter randomized trial, providing Level 1 evidence for digital pain interventions (Gendreau et al., 2024). Emerging evidence also supports novel digital modalities; telehealth-delivered virtual reality interventions have demonstrated significant reductions in chronic pain intensity, anxiety, and pain catastrophizing in a randomized crossover trial (Colloca et al., 2025). Digital CBT for insomnia (dCBT-I), a common comorbidity of chronic pain, has also been shown to be effective when added to standard chronic pain treatment, significantly improving sleep and positively impacting pain-related disability (Burns et al., 2024; Olsen et al., 2025).

Specifically for older populations, a systematic review and meta-analysis found moderate positive effects of digital pain management interventions on pain outcomes in older adults, while highlighting the need for age-appropriate design features (Silva A. G. et al., 2025; Silva M. T. et al., 2025). A scoping review further confirmed that technology-delivered multimodal programs can improve both pain and function when designed with geriatric-specific considerations (De Lucia et al., 2024). For these platforms to be successful in the geriatric population, user-centered design is critical, requiring simplified interfaces, large and clear text, options for multimodal content delivery (text, audio, video), and robust, easily accessible technical support (Taygar et al., 2025).

However, critical examination reveals significant concerns about exacerbating health disparities. The “digital divide”—unequal access to technology, internet connectivity, and digital literacy—disproportionately affects the very populations most burdened by chronic pain: older adults, low-income individuals, racial/ethnic minorities, and rural residents (Hepburn et al., 2025). Research has found that the digital health literacy of elderly patients with chronic diseases is generally low, influenced by multiple personal and social factors including cognitive limitations, challenges of digital adaptation, and concerns about digital security risks (Shao et al., 2025). Addressing these barriers through community-based digital literacy programs, caregiver-assisted platforms, and hybrid delivery models will be essential to realizing the potential of digital therapeutics for geriatric pain management.

### 7.3 Modulating perception: hypnosis and mindfulness-based therapies

Hypnosis: Clinical hypnosis is a state of focused attention and heightened suggestibility that can be used to alter the perception of pain and promote relaxation. Systematic reviews suggest that clinical hypnosis may help reduce chronic pain, particularly when combined with patient education (Jones et al., 2024). During hypnosis, a therapist can guide the patient to experience sensations of warmth, numbness, or coolness in the painful area, or to reframe the pain as a neutral sensation. For older adults, effective hypnotic communication requires clear, patient-centered language and consideration of their unique educational and cognitive needs (Jensen and Patterson, 2014; Sheikh et al., 2025).

Mindfulness-Based Therapies: Rooted in ancient contemplative traditions, mindfulness-based therapies, such as Mindfulness-Based Stress Reduction (MBSR), teach patients to pay attention to their pain and other sensations in a non-judgmental, accepting way. The goal is not to eliminate the pain, but to uncouple the raw sensory information from the emotional reaction and suffering that usually accompanies it. By observing the pain as a fluctuating, impersonal sensation, patients can reduce the emotional distress and disability associated with it. A randomized controlled trial found that ultrasound-guided acupotomy combined with mindfulness meditation was significantly more effective than acupotomy alone or medication in reducing pain, improving function, and alleviating emotional and sleep disturbances in patients with lumbar MPS (Liu et al., 2025).

By integrating these evidence-based psychological interventions, clinicians can empower older adults with the skills to manage not just the physical, but also the profound emotional and psychological burden of chronic myofascial pain.

## 8 Integrative treatment and the multidisciplinary collaboration model

The complexity of geriatric MPS, as detailed in the preceding sections, makes it clear that no single intervention, whether a pill, an injection, or a specific therapy, can fully address all dimensions of the condition. The most effective, evidence-based, and patient-centered approach is one that is integrative and multidisciplinary. An integrative model of care moves beyond simply co-locating different services; it involves the active coordination and synthesis of multiple evidence-based modalities into a single, cohesive treatment plan that is tailored to the individual patient’s unique biopsychosocial profile (den van Block et al., 2025).

### 8.1 The core multidisciplinary team (MDT)

An effective MDT for geriatric pain management should be built around a core team of professionals who bring complementary expertise. The composition of the team may vary based on resources, but an ideal team would include:

Geriatrician or Primary Care Physician: Serves as the team coordinator, managing the patient’s overall health, comorbidities, and coordinating care among specialists.

- Pain Medicine Specialist: Provides expertise in advanced diagnostic techniques (e.g., ultrasound) and interventional procedures (e.g., ultrasound-guided injections, neuromodulation).
- Physical Therapist: Designs and supervises individualized therapeutic exercise programs, performs manual therapy, and educates the patient on posture and body mechanics.
- Clinical Psychologist or Behavioral Health Specialist: Delivers evidence-based psychological therapies (CBT, ACT), teaches coping skills, and addresses comorbid mental health conditions.
- Clinical Pharmacist: Plays a crucial role in medication management, particularly in conducting comprehensive medication reviews and leading the de-prescribing process.
- Social Worker or Care Coordinator: Helps to address social determinants of health, connects the patient with community resources, and helps communication among the patient, family, and healthcare team.

### 8.2 The MDT in action: a real-world case example

The genesis of this review lies, in part, in a deeply personal clinical experience that underscores both the devastating impact of geriatric MPS and the critical need for integrated, multidisciplinary care. One of the authors (anonymized for privacy) witnessed firsthand the challenges faced by a 74-year-old woman—a close family member—whose case exemplifies the complex, multifactorial nature of MPS in older adults and the profound gaps that exist in current care delivery, particularly in resource-limited settings.

#### 8.2.1 Clinical presentation and cascade of events

The patient, with a longstanding history of type 2 diabetes, hypertension, and severe osteoporosis, underwent endovascular intervention in August 2024 for critical limb ischemia secondary to peripheral arterial disease and diabetic foot complications. Post-procedure, she was discharged home with apparent initial recovery. However, within days, and without any identifiable trauma or precipitating event, she sustained a spontaneous L1 vertebral compression fracture—a stark reminder of the fragility inherent in advanced osteoporosis. She was readmitted and underwent percutaneous vertebroplasty (bone cement augmentation), which provided significant relief from the acute vertebral pain.

Yet, in the weeks following vertebroplasty, a new and debilitating problem emerged: severe myofascial pain syndrome affecting the lumbar paraspinal muscles. Despite resolution of the fracture-related pain, she developed profound functional impairment. For 2–3 consecutive months, she was unable to independently rise from a lying to sitting position or to get out of bed without assistance, although once upright, she could walk with relatively preserved gait. The myofascial pain, characterized by palpable trigger points, muscle stiffness, and exquisite tenderness, became the primary barrier to her functional independence and quality of life.

#### 8.2.2 Multidisciplinary intervention: strengths and limitations

Recognizing the complexity of her condition, a rehabilitation medicine physician coordinated care involving physical therapists and acupuncturists. The team used a multimodal approach:

Physical Therapy: Gentle mobilization, therapeutic exercises targeting core stability and lumbar flexibility, and progressive functional training.

Acupuncture: Traditional Chinese acupuncture targeting local trigger points and broader pain modulation pathways.

Family Support: Family members, who were healthcare professionals (though not specialized in geriatric pain or rehabilitation), provided continuous psychological support, reassurance, and assistance with activities of daily living.

This coordinated effort, while valuable, also revealed significant systemic gaps:

Absence of Specialized Psychological Support: Despite clear signs of fear-avoidance behavior, anxiety related to recurrent falls or fractures, and possible depression secondary to prolonged disability, no psychologist or mental health professional was integrated into the care team. In the Chinese healthcare system, as in many other settings, geriatric psychology and psychosocial support remain underdeveloped and largely inaccessible.

Lack of Social Work Services: There was no formal assessment of social determinants of health, caregiver burden, or community resources. Social workers, who play a critical role in MDT models in high-income countries, are rarely part of geriatric care teams in China.

Despite these limitations, gradual improvement occurred over several months, driven largely by the persistence of the rehabilitation team and the unwavering support of family caregivers. However, the prolonged duration of disability, the emotional toll on both patient and family, and the lack of a truly integrated, patient-centered MDT approach left a lasting impression.

#### 8.2.3 Reflections and the rationale for this review

This case is not unique. It reflects a broader reality: older adults with MPS, particularly those with multimorbidity and frailty, face a cascade of biological, psychological, and social challenges that no single clinician or discipline can adequately address in isolation. The experience of caring for this patient—and witnessing the gaps in care—served as a powerful catalyst for the present review.

The core questions that emerged were:

- What does the evidence tell us about the optimal management of MPS in older adults?
- How can we integrate precision geriatrics, digital therapeutics, and structured deprescribing into a cohesive, practical framework?
- What lessons can be drawn from high-resource settings to inform care delivery in resource-limited contexts?
- How can we move from fragmented, siloed care to truly integrated, multidisciplinary, patient-centered models?

### 8.3 Toward clinical translation: a stepwise implementation pathway

Drawing on the lessons from this case and the evidence synthesized throughout this review, we propose a practical, stepwise implementation pathway that can be adapted to diverse healthcare settings and resource levels (Table 1).

**Table 1:** Stepwise clinical implementation pathway for geriatric myofascial pain syndrome.

Step	Objective	Key tools/assessments	Applicable settings
1. Screen and Identify	Routine screening for MPS in older adults with chronic musculoskeletal pain	Systematic palpation for MTrPs; SARC-F (sarcopenia); Clinical Frailty Scale; GDS; PCS	Primary care, geriatric clinics
2. Comprehensive Multidimensional Assessment	Objective characterization of trigger points and comprehensive geriatric evaluation	Well-resourced: Ultrasonography with elastography, CGA (TUG, gait speed, vitamin D, cognitive screening) Resource-limited: Clinical palpation + standardized functional assessments	Rehabilitation centers, specialist clinics; adaptable to community settings
3. Risk Stratification and Treatment Planning	Categorize patients to guide treatment intensity	Low-risk: Exercise + self-management education Moderate-risk: Multimodal (exercise, manual therapy, psychological support) + medication review High-risk: Full MDT + intensive de-prescribing + supervised rehabilitation	All settings; complexity of intervention scaled to resources
4. Implement Multimodal Treatment	Non-pharmacological interventions first; precision prescribing for pharmacological agents	Exercise (FITT-VP principles); CBT/ACT (face-to-face, telehealth, or digital); De-prescribing protocols for PIMs	Multidisciplinary teams; digital platforms for remote delivery
5. Monitor, Reassess, and Adapt	Track outcomes at 2 weeks, 1 month, 3 months; dynamically adjust treatment	NRS (pain); TUG, gait speed (function); Patient-reported outcomes (QoL, self-efficacy)	All settings; telehealth for remote monitoring

MTrPs, myofascial trigger points; SARC-F, Strength, Assistance with walking, Rising from a chair, Climbing stairs, Falls; GDS, Geriatric Depression Scale; PCS, Pain Catastrophizing Scale; CGA, comprehensive geriatric assessment; TUG, Timed Up and Go; MDT, multidisciplinary team; FITT-VP, Frequency, Intensity, Time, Type, Volume, Progression; CBT, cognitive behavioral therapy; ACT, acceptance and commitment therapy; PIMs, potentially inappropriate medications; NRS, Numeric Rating Scale; QoL, quality of life.

Step 1—Screen and Identify: In primary care or geriatric clinic settings, clinicians should incorporate routine screening for MPS in older adults presenting with chronic musculoskeletal pain. This includes systematic palpation for myofascial trigger points combined with validated screening tools for sarcopenia (SARC-F questionnaire), frailty (Clinical Frailty Scale), and psychosocial risk factors (Geriatric Depression Scale, Pain Catastrophizing Scale).

Step 2—Comprehensive Multidimensional Assessment: For patients identified with suspected MPS, a comprehensive assessment should be conducted. In well-resourced settings, this includes musculoskeletal ultrasonography with elastography for objective trigger point characterization, alongside comprehensive geriatric assessment (CGA) encompassing functional mobility (Timed Up and Go, gait speed), nutritional status (vitamin D levels), and cognitive screening. In resource-limited settings, clinical palpation combined with standardized functional assessments can provide an adequate baseline.

Step 3—Risk Stratification and Treatment Planning: Based on assessment findings, patients should be stratified into risk categories to guide treatment intensity. Low-risk patients (preserved function, minimal psychosocial burden, limited polypharmacy) may be managed primarily with exercise prescription and self-management education. Moderate-risk patients (emerging functional decline, mild-to-moderate psychosocial distress, polypharmacy) require multimodal treatment combining exercise, manual therapy, and psychological support, with concurrent medication review. High-risk patients (significant frailty, severe psychosocial burden, complex polypharmacy) warrant full multidisciplinary team involvement with intensive de-prescribing, supervised rehabilitation, and specialist psychological intervention.

Step 4—Implement Multimodal Treatment with Non-Pharmacological Priority: Regardless of risk category, non-pharmacological interventions should be initiated first. Exercise prescription should follow FITT-VP principles with geriatric adaptations. Psychological interventions (CBT or ACT) should be offered through the most accessible modality—face-to-face, telehealth, or digital platforms—based on patient preference and digital literacy. Pharmacological interventions should follow precision prescribing principles, with concurrent initiation of de-prescribing protocols for potentially inappropriate medications.

Step 5—Monitor, Reassess, and Adapt: Treatment response should be monitored at regular intervals (2 weeks, 1 month, 3 months) using standardized outcome measures including pain intensity (Numeric Rating Scale), functional status (TUG, gait speed), and patient-reported outcomes (quality of life, self-efficacy). Treatment plans should be dynamically adjusted based on response, with escalation or de-escalation of interventions as clinically indicated.

This stepwise pathway is designed to be scalable: the core principles (screen, assess, stratify, treat non-pharmacologically first, monitor) can be implemented even in settings without access to advanced imaging or full multidisciplinary teams, while the framework accommodates the integration of precision diagnostics and digital therapeutics as resources allow.

## 9 Limitations of the current framework

While this review proposes an innovative geroscience-informed framework for managing myofascial pain syndrome in older adults, several limitations must be acknowledged. First, from a conceptual standpoint, the integration of multiple aging mechanisms—including sarcopenia, inflammaging, cellular senescence, and mitochondrial dysfunction—into a unified clinical framework remains largely theoretical. The relative contributions and complex interactions among these mechanisms in the pathogenesis of geriatric MPS are not yet fully elucidated, and their weighting in individual patients may vary considerably. Second, from an evidence perspective, many of the innovative diagnostic and therapeutic approaches discussed in this review—such as elastography-based trigger point assessment, digital therapeutics platforms, and senolytic interventions—lack high-quality clinical evidence, particularly randomized controlled trials, specifically in elderly populations with MPS. Much of the supporting evidence is extrapolated from younger populations or related conditions. Third, from an implementation standpoint, the proposed framework requires access to multidisciplinary teams, advanced imaging technologies, and digital health infrastructure that may not be available in resource-limited settings. The digital divide and varying levels of digital health literacy among older adults present additional barriers to the widespread adoption of digital therapeutics. Future research should aim to validate the individual components of this framework through rigorous clinical trials and develop implementation strategies that are adaptable to diverse healthcare contexts.

## 10 Conclusions and future directions

The clinical case described in Section 8.2 illustrates a reality faced by many older adults worldwide: myofascial pain syndrome in the elderly is not merely a localized musculoskeletal complaint, but a complex, multifactorial condition intertwined with frailty, multimorbidity, psychological distress, and systemic gaps in care delivery. Addressing this challenge demands a paradigm shift toward a holistic, patient-centered, biopsychosocial framework grounded in the principles of geroscience.

This review has outlined such a framework by integrating precision geriatrics, digital therapeutics, and structured de-prescribing into a comprehensive model of care. The key tenets of this framework include recognizing age-specific pathophysiological mechanisms such as sarcopenia, mitochondrial dysfunction, inflammaging, cellular senescence, and central sensitization; employing multidimensional assessment encompassing imaging, comprehensive geriatric assessment, functional evaluation, and psychosocial screening; prioritizing non-pharmacological interventions—including movement therapies, adapted manual therapy, and psychological interventions—as first-line treatments; implementing precision prescribing alongside proactive de-prescribing to minimize iatrogenic harm; and fostering integrative, multidisciplinary collaboration in which communication is seamless, goals are shared, and the patient remains at the center.

Despite the strength of this framework, significant gaps remain between current evidence and clinical practice. Future research should prioritize large-scale, geriatric-specific randomized controlled trials that measure outcomes most meaningful to older adults, including functional independence, quality of life, and avoidance of harm. Equally important is the development of biomarkers to enable earlier diagnosis and more targeted therapeutic approaches. Comparative effectiveness research is needed to guide clinical decision-making and resource allocation across diverse settings, while implementation science will be essential to translate evidence-based frameworks into routine clinical care. Throughout this process, meaningful engagement of patients and caregivers must be ensured so that interventions remain acceptable, feasible, and aligned with individual values and preferences.

Realizing this vision will require concerted effort across multiple stakeholders. Clinicians are encouraged to adopt a biopsychosocial lens, prioritize non-pharmacological approaches, and engage in interdisciplinary collaboration. Policymakers must invest in the infrastructure necessary to support multidisciplinary, team-based geriatric care. Researchers should ensure that the most vulnerable older adults are adequately represented in clinical studies. Behind every clinical guideline and systematic review are real individuals whose suffering is profound and whose need for compassionate, evidence-based care is urgent. By embracing the integrated approach outlined in this review, we hope to move closer to alleviating the burden of myofascial pain and improving the function, independence, and quality of life of older adults worldwide.
